# Isolated Liver Metastasis of Sacrococcygeal Chordoma: Case Report and Review of the Literature

**DOI:** 10.1155/2014/826584

**Published:** 2014-06-11

**Authors:** Murat Akyol, Umut Varol, Ibrahim Yildiz, Ibrahim Vedat Bayoglu, Yasar Yildiz, Lutfiye Demir, Ahmet Dirican, Alper Can, Suna Cokmert, Mine Tunakan Oztop, Ahmet Alacacioglu, Yuksel Kucukzeybek, Mustafa Oktay Tarhan

**Affiliations:** ^1^Department of Medical Oncology, Izmir Kâtip Çelebi University, Ataturk Training and Research Hospital, Medical Oncology Clinic, 35360 Izmir, Turkey; ^2^Department of Pathology, Izmir Kâtip Çelebi University, Ataturk Training and Research Hospital, 35360 Izmir, Turkey; ^3^Institute of Oncology, Dokuz Eylül University, 35360 Izmir, Turkey

## Abstract

Chordomas are rare neoplasms arising from notochordal remnants and may develop anywhere in the body while the most common anatomic site is the sacrococcygeal area. The most effective treatment of chordoma is surgery. Chordomas rarely metastasize to lung, bone, soft tissue, liver, lymph nodes, and skin. However, there is currently no standard systemic treatment for advanced stage chordoma. Here, we reported a rare presentation of chordoma patient with liver only metastases and poor prognosis.

## 1. Introduction

Chordomas are extremely rare primary malignant bone tumors that arise from notochordal remnants. They are usually seen in adults and occur in the fifth to seventh decades of life [1]. The most common anatomic site is the sacrococcygeal area (50–60%). These tumors may also develop in the sphenooccipital (35%) and cervical (15%) regions [2]. Chordomas arising in the vertebral bodies have a poorer prognosis than those in the other sites [3]. These tumors tend to invade adjacent structures, while they rarely metastasize to distant sites [4]. Though rare, metastases of chordoma can arise even when the tumor was adequately resected. Here, we reported a rare presentation of chordoma patient with liver only metastases and poor prognosis.

## 2. Case Presentation 

In May 2009, a 62-year-old male patient was admitted with complaints of severe pain and edema in gluteal region. He had a history of cholecystectomy and pulmonary tuberculosis 3 years before. Pelvic computed tomography (CT) revealed a huge mass (15∗13 cm) infiltrating the coccyx and most of the sacrum without any regional lymphadenopathies. Chordoma of the sacrococcygeal area was diagnosed by Tru-Cut biopsy under radiological evaluation. In June 2010, the chordoma in the sacral region was excised with tumor-free margins. Afterwards, adjuvant radiotherapy was applied for about 30 days; pelvic box tumor total dose: 50 gray in 25 fractions and boost tumor total dose: 10 gray in 5 fractions. After completion of the treatment, the patient was followed up 10 Gy boost dose was applied after 50 Gy to the total dose of 60 Gy in 30 fractions. 2 Gy was applied per fraction every weekdays.

**Figure 1:**
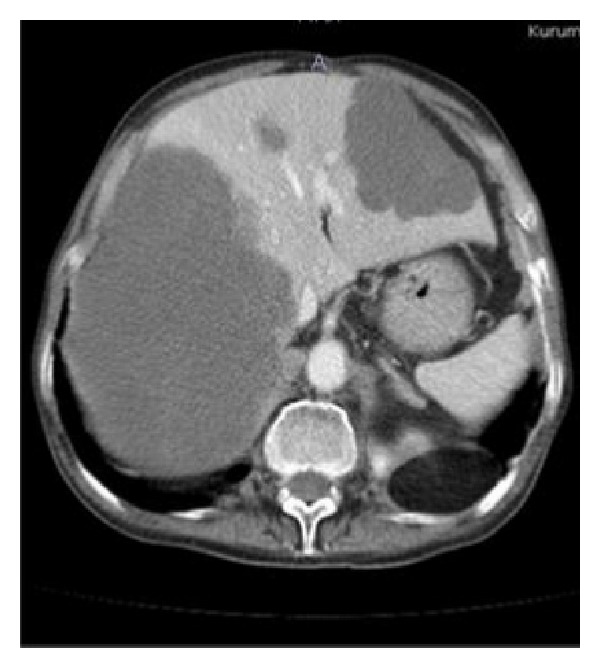
Abdominal computerized tomography of the patient that shows multiple hepatic metastases.

**Figure 2:**
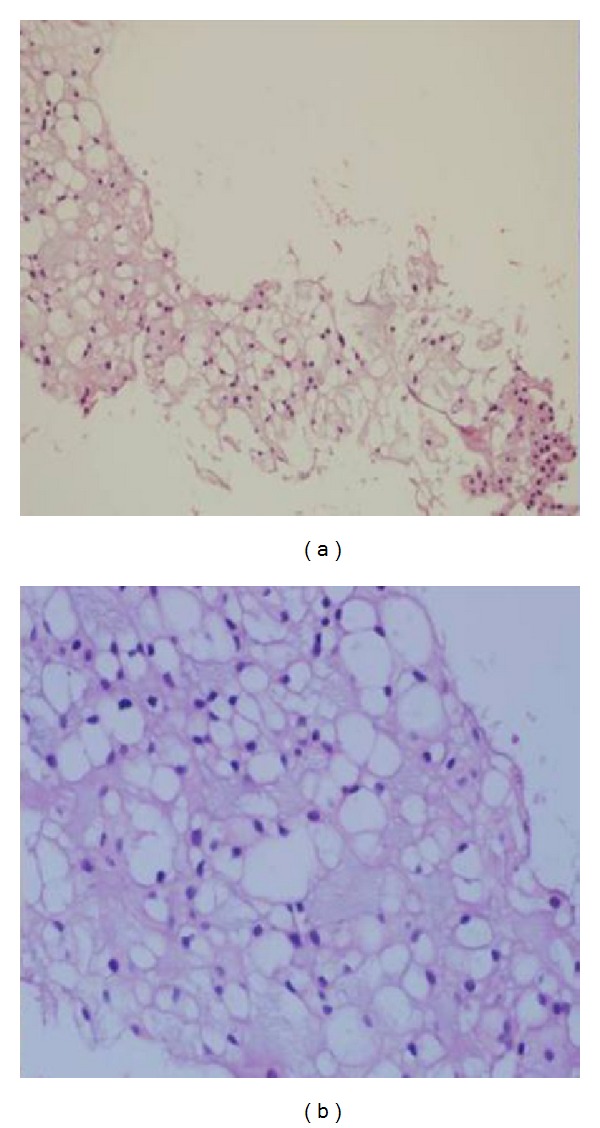
(a) Tumor cells with large, pellucid, and eosinophilic cytoplasm in the myxoid matrix (hematoxylin and eosin stain ×100). (b) Physaliphorous cells with large vacuoles in cytoplasm that show different forms and nuclear size (hematoxylin and eosin stain ×400).

A local tumor recurrence developed after about two years and required additional surgical procedures. The patient was reoperated for its tumor recurrence. Soon after the operation, during routine examination, liver function tests were found moderately high. Abdominal computerized tomography (CT) revealed multiple hypodense lesions in the liver: 17 cm mass in the right lobe, about 7 cm mass in the left lobe, and 3 cm mass in junction of the right and the left lobe of the liver (Figure 1). Therefore, we decided to proceed fine needle aspiration of the lesion under ultrasound guidance. A histological examination of the biopsy from the liver lesion showed a metastatic focus from the sacrococcygeal chordoma (Figures 2(a) and 2(b)). We then decided to treat our patient with imatinib mesylate. However, the patient died because of hepatic failure before starting the treatment.

## 3. Discussion

Chordomas are very rare tumors arising from remnants of the primitive notochord and account for 1 to 4% of all primary malignant bone neoplasms [1]. These tumors often result in people aged 30–60 and tend to affect males more than females [3]. Chordomas are usually low-grade tumors and grow slowly [5]. According to microscopic morphology, there are 3 different types: conventional, chondroid, and dedifferentiated [6]. Less than 5% of all patients may have an aggressive “dedifferentiated” high-grade variety [5]. Dedifferentiated chordoma is a rare and aggressive variant of the conventional chordoma in which an area undergoes transformation to a high-grade lesion and affects overall survival [7].

Chordomas are locally aggressive and usually recur at the region of their origin. Heffelfinger et al. determined that the first local recurrence of these tumors resulted in most of the patients within 2 years of initial presentation in vertebral chordomas, 2.5 years for sacrococcygeal chordomas, and 3 to 4 years for sphenooccipital chordomas [8].

Chordomas can rarely metastasize to distant sites and the risk of metastasis for chordomas is very high after radiation therapy. Most of the patients metastasize to lungs (58%) followed by 33% to the lymph nodes, 22% to the liver, 17% to bone, and 9% to skeletal muscle [9]. The sacrococcygeal chordomas have been reported to metastasize more frequently than the other types [1].

Many histological studies have been carried out to determine the factors that would estimate the metastatic potential of a chordoma. Predicting factors for the metastatic potential of a chordoma include lack of intercellular material, variable cellular and nuclear size, increased mitosis, cell density, excessive intracellular mucin formation, pleomorphism, anaplasia, hyperchromatism, and invasiveness of tumor. However, these factors have also been shown in nonmetastatic chordomas. The accurate diagnosis depends on histological examination together with ultrastructure and immunohistological studies. The cytopathological hallmarks of chordomas contain dissociated and small groups of polygonal cells, along with presence of physaliphorous cells in a background of conspicuous extracellular myxoid matrix [9].

The most effective treatment of chordoma is surgery. It is usually difficult to obtain tumor-free margins at initial surgery, because the anatomical location of these tumors limits the ability of the surgeon to remove the entire tumor [10]. The use of primary or adjuvant radiotherapy is controversial due to its radioresistance. Despite minimal response to radiotherapy, many authors use radiotherapy for some situations including local control, inoperable disease, contaminated surgical margins, or elderly patients [11]. In addition, there is currently no standard systemic therapy for advanced stage chordoma. Chemotherapy regimens including anthracyclines, cisplatin, and alkylating agents have been used in advanced chordoma and have not been shown to be effective [12]. Intratumoral chemotherapy is an alternative therapeutic option to systemic treatment and in combination with surgical treatment may improve the local control rate [13]. Casali et al. showed presence of the PDGF receptor on chordoma cell membranes. Among molecular targeted therapies, imatinib, an inhibitor of platelet-derived growth factor receptor-*β* and c-KIT, has been shown to be active in the treatment of advanced chordoma [14]. The epidermal growth factor receptor (EGFR) antagonists have also been shown to be effective in patients with chordoma refractory to imatinib. In the literature, there are two cases with advanced chordoma treated with the gefitinib plus cetuximab, while the other two patients were treated with erlotinib alone [15].

We presented this case due to its uncommon presentation with isolated liver metastasis. In the literature, there is only one chordoma patient that had isolated liver metastasis. Soon after the diagnosis of metastases, the patient died which means that close follow-up of the high-risk chordoma patients is very important even after complete resection of the primary tumor. Although a lot of new molecular targeted therapies are used for advanced stage chordoma, there is currently no standard systemic treatment. Chordoma with liver metastasis still remains poor prognosis and we need new therapeutic approaches for its treatment.
